# A nationwide survey on the management of the COVID-19 pandemic and respiratory disease in South Korea

**DOI:** 10.3389/fmed.2022.965651

**Published:** 2022-09-23

**Authors:** Lin Ang, Mi Hong Yim, Eunhye Song, Hye Won Lee, Hyangsook Lee, Tae-Hun Kim, Merlin Willcox, Xiao-Yang Hu, Joelle Houriet, Bertrand Graz, Je-Won Lee, Yunho Jang, Jung Tae Kim, Eunsop Kim, Yong Hee Park, Myeong Soo Lee

**Affiliations:** ^1^KM Science Research Division, Korea Institute of Oriental Medicine, Daejeon, South Korea; ^2^Digital Health Research Division, Korea Institute of Oriental Medicine, Daejeon, South Korea; ^3^Global Cooperation Center, Korea Institute of Oriental Medicine, Daejeon, South Korea; ^4^KM Convergence Research Division, Korea Institute of Oriental Medicine, Daejeon, South Korea; ^5^Korean Medicine Convergence Research Information Center, College of Korean Medicine, Kyung Hee University, Seoul, South Korea; ^6^Department of Clinical Korean Medicine, Graduate School, Kyung Hee University, Seoul, South Korea; ^7^School of Primary Care, Population Sciences and Medical Education, Faculty of Medicine, University of Southampton, Southampton, United Kingdom; ^8^Antenna Foundation, Geneva, Switzerland; ^9^BM Korean Internal Medicine Clinic, Daegu, South Korea; ^10^Changpo Kyunghee Clinic, Pohang, South Korea; ^11^I-MOM Korean Medicine Clinic, Jeju, South Korea; ^12^You and Green Korean Medical Clinic, Daejeon, South Korea; ^13^You and Green Korean Medical Clinic, Busan, South Korea

**Keywords:** COVID-19, behavior, public health, social measures, wellbeing, health care

## Abstract

**Background:**

This study aimed to explore individual prevalence of respiratory symptoms and to describe the Korean population's treatment approaches, preventive health behaviors, and mental health conditions during the pandemic.

**Methods:**

We analyzed responses from an online nationwide survey, conducted between February 2021 to May 2021, about people's experiences during the pandemic. Statistical analysis was also performed to see if there were any significant differences in treatment and prevention strategies between different groups of respondents (between those had respiratory symptoms, compared with those who did not, and between those tested positive for COVID-19, compared with those who did not).

**Results:**

A total of 2,177 survey respondents completed the survey and, of these, only 142 had experienced symptoms. The most frequently reported respiratory infections related symptoms were runny or blocked nose (47.6%), cough (45.5%), fever (44.1%), sore throat (42.0%), and fatigue (30.1%). More than half of the respondents (53.1%) used complementary and alternative medicine (CAM) approaches as means of preventive measures. In terms of preventive behaviors, the more emphasized behaviors were mask-wearing (58.9%) and hand-washing after coming home (42.7%). The majority of the respondents (64.9%) did not show signs of mental health issues.

**Conclusion:**

In South Korea, conventional medicine was mainly used for COVID-19 treatment whereas CAM was commonly used as preventive measures. COVID-19 was also found to have less impact on the general population's mental health. The findings of this study may shed light on how the pandemic impacted the general population.

## Introduction

The coronavirus disease 2019 (COVID-19) is a recent pandemic which highly impacts the public health and wellbeing (1–3). The severity of disease varies greatly from person to person, ranging from asymptomatic to mild flu-like symptoms to multiorgan failure, and even to death (4). Despite vaccination, non-pharmaceutical interventions including social distancing, putting on mask, and quarantine are still being implemented in many countries to prevent the widespread outbreaks of COVID-19. In the meantime, the pandemic response is still focusing on reducing hospitalization and fatality for those susceptible to COVID-19.

During the initial phase of the pandemic, South Korea was considered to have a well-organized epidemic control where various measures were taken to limit the spread of outbreak including mass population testing, tracing and isolating close contacts, and mandatory mask-wearing (5, 6). The government has also tried to contain the outbreak without a total lockdown of the country by imposing social distancing measures (7). The vaccination of COVID-19 began at the end of August 2021. Despite the efforts, the number of COVID-19 infections remains high.

Individuals' health-related behavior and lifestyle have changed significantly due to COVID-19 (8). Amid the COVID-19 outbreak, people began to engage in the recommended preventive health behaviors such as mask mandates, physical distancing, washing hands, and disinfecting surfaces (9). Social distancing, restrictions on daily activities and social gatherings, and closure of schools and universities have highly impacted the mental health of general population. On the other hand, COVID-19 produces similar symptoms to other viral respiratory infections (10). While most viral respiratory infections cause a relatively mild disease, COVID-19 is potentially much more serious and requires more attention. As there are many crossover symptoms between COVID-19 and respiratory infections (11, 12), it is important to make a conclusive diagnosis through proper testing.

In order to control the pandemic, the epidemiological community turned their attention to evaluating measures that might help contain the virus while vaccinations and pharmaceutical interventions were being developed. While there are many studies conducted on vaccinations and clinical manifestations of COVID-19, preventive studies have been limited to those conducted in China, United States, United Kingdom, Australia, Netherlands, Germany, and Italy (13–16). Several reviews also highlighted the benefits of herbal medicine, physical activity, and dietary supplements as a complement for the treatment and prevention of COVID-19 (17–20).

In light of these considerations, a national survey was conducted to explore individual prevalence of respiratory symptoms and to describe the Korean population's treatment approaches, preventive health behaviors, and mental health conditions during the pandemic.

## Methods

### Study design and setting

A nationwide survey, conducted *via* online Limesurvey platform in February 2021, was translated and modified from an international retrospective survey led by a team of researchers from University of Southampton and University of Geneva. The original survey was modified and localized by a research team from Korea Institute of Oriental Medicine and Kyunghee University to ensure that the translated survey was on-target, contextually precise, and culturally correct. The translated version was piloted to test the survey functionalities and to refine the whole survey before starting actual data collection. The survey was then disseminated to the general public within Korea using social media and online advertisement by means of convenience sampling and snowball sampling techniques. This study received ethical approval from Kyunghee University [KHSIRB-20-574 (EA), November 2020] and University of Southampton (ERGO 56975, May 2020). The STrengthening the Reporting of OBservational studies in Epidemiology (STROBE) guidelines were followed for reporting this study (21).

### Participants

All members of the public aged 16 and above were eligible to participate in this survey. Survey participants were required to provide informed consent for the use of their data at the start of the survey. Those who were under the age of 16 or lacked mental capacity to consent to the survey were excluded.

### Variables and bias

Respondents were asked about the presence and duration of COVID-19 symptoms, the type of treatment they received during their initial COVID-19 illness (e.g., conventional medicine or other approaches), actions taken as risk-reduction measures (e.g., vaccination, masking, social distancing etc.), type of approaches attempted to prevent COVID-19 and impact of the pandemic on mental health and wellbeing. The mental health status of the respondents was investigated using Patient Health Questionnaire for Depression and Anxiety (PHQ-4), which is a composite 4-item scale consisting of two core criteria for each depressive and anxiety disorder (22). PHQ-4 was used in order to examine both anxiety and depression due to COVID-19.

As this study was a retrospective survey, it may be subject to self-reporting bias and recall bias. To minimize the potential bias of this study, the survey questions were carefully designed and constructed. As the length of the recall period can affect data accuracy, the length of the recall period was also fairly short since the survey was conducted during early pandemic. Besides, a reminder was given to the survey respondents at the beginning of the survey if they had any memory aids such as medication diaries to enhance recall and reduce under-reporting.

### Statistical methods

Statistical analyses were performed using R Statistical Software (v4.1.2; R Core Team 2021. R: A language and environment for statistical computing. R Foundation for Statistical Computing, Vienna, Austria. https://www.R-project.org/). Demographic data and information about pre-existing health conditions were collated and analyzed descriptively. Statistical analysis was also carried out to investigate any differences in treatment and preventive approaches between those had respiratory symptoms, compared with those who did not, and between those who tested positive for COVID-19, compared with those who did not. Fisher's exact test was used to examine between-group differences, with *p* < 0.05 taken to indicate statistical significance. The most recent data was used for analysis when there were duplicate entries.

## Results

### General characteristics of survey respondents

There were 3,188 responses between 9 February 2021 and 3 May 2021 which, excluding 980 partial entries and 31 duplicates, left a final sample of 2,177 for analysis (Table 1). The represented mean age was 32.2 years and 1,922 (88.3%) had no pre-existing health issues. A total of 68 (3.1%) were tested positive for COVID-19 and only 17 (11.9%) presented respiratory infections related symptoms.

**Table 1:** Demographics of survey respondents.

**Category**	**Total (*n*, %)**	**Without symptoms (*n*, %)**	**With symptoms (*n*, %)**
**Gender**			
Female	1,195 (54.9)	1,123 (55.2)	72 (50.4)
Male	976 (44.8)	906 (44.5)	70 (49.0)
Non-binary	6 (0.3)	5 (0.3)	1 (0.7)
**Age (mean** **±SD, yrs)**	32.21 ± 8.51	32.11 ± 8.41	33.62 ± 9.73
**Height (mean** **±SD, cm)**	167.45 ± 8.13	167.35 ± 8.09	168.77 ± 8.56
**Weight (mean** **±SD, kg)**	62.79 ± 16.86	62.63 ± 17.05	64.87 ± 14.09
**Long-standing health issue**			
No health issues	1,922 (88.3)	1,807 (88.8)	115 (80.4)
Hypertension	35 (1.6)	30 (1.5)	5 (3.5)
Diabetes	21 (1.0)	16 (0.8)	5 (3.5)
Chronic obstructive pulmonary disease	3 (0.1)	1 (0.1)	2 (1.4)
Asthma	20 (0.9)	13 (0.6)	7 (4.9)
Heart/blood vessels-related conditions	5 (0.2)	5 (0.3)	0 (0)
Cancer (On active treatment)	2 (0.1)	2 (0.1)	0 (0)
Cancer (Not on active treatment)	10 (0.5)	6 (0.3)	4 (2.8)
Liver disease	4 (0.2)	3 (0.2)	1 (0.7)
Mental health issues	52 (2.4)	45 (2.2)	7 (4.9)
**COVID-19 polymerase chain reaction (PCR) test**			
Not tested	1,630 (74.9)	1,561 (76.8)	69 (48.3)
Tested negative	464 (21.3)	407 (20.0)	57 (39.8)
Tested positive	68 (3.1)	51 (2.5)	17 (11.9)
Unsure	15 (0.7)	15 (0.7)	0 (0)
**Duration of symptoms when testing**			
No symptoms	358 (67.4)	343 (75.1)	15 (20.3)
< 7 days	123 (23.2)	73 (16.0)	50 (67.6)
7–14 days	36 (6.8)	29 (6.4)	7 (9.5)
More than 14 days	14 (2.6)	12 (2.6)	2 (2.7)
Not reported	1,646	1,577	69
**COVID-19 antibody test**			
Not tested	2,009 (92.3)	1,895 (93.2)	114 (79.7)
Tested positive	53 (2.4)	46 (2.3)	7 (4.9)
Tested negative	80 (3.7)	67 (3.3)	13 (9.1)
Unsure	35 (1.6)	26 (1.3)	9 (6.3)
**Full time education (including postgraduates)**	16.26 ± 2.92	16.28 ± 2.87	16.06 ± 3.61
**Financial concerns during the pandemic**			
None	940 (43.3)	879 (43.3)	61 (43.3)
Concerning	1,031 (47.5)	964 (47.5)	67 (47.5)
Very concerning	131 (6.0)	120 (5.9)	11 (7.8)
Extremely concerning	67 (3.1)	65 (3.2)	2 (1.4)
Not reported	8	6	2
**Received influenza/flu vaccine**			
Yes, in 2020	626 (28.8)	567 (27.9)	59 (41.3)
Yes, in 2019	442 (20.3)	402 (19.8)	40 (28.0)
No	293 (13.5)	274 (13.5)	19 (13.3)
Don't know	984 (45.2)	935 (46.0)	49 (34.3)
**Job**			
Full-time student	425 (19.6)	397 (19.6)	28 (19.6)
Working outside of home	874 (40.2)	809 (39.9)	65 (45.5)
Working from home	285 (13.1)	269 (13.3)	16 (11.2)
Working partial remote/hybrid	206 (9.5)	202 (10.0)	4 (2.8)
Retired	39 (1.8)	34 (1.7)	5 (3.5)
Furloughed	137 (6.3)	126 (6.2)	11 (7.7)
Unemployed	143 (6.6)	134 (6.6)	9 (6.3)
Other (freelancer, part-time employee, volunteer, etc.)	64 (3.0)	59 (2.9)	5 (3.5)
Not reported	4	4	0
**Living alone or with someone**			
Living alone	1,210 (55.8)	1,158 (57.1)	52 (36.4)
Living with other people (family, housemates, residences, etc.)	960 (44.2)	869 (42.9)	91 (63.6)
Not reported	7	7	0
**Alcohol consumption**			
Never	829 (38.1)	787 (38.7)	42 (29.4)
Monthly or less	673 (30.9)	632 (31.1)	41 (28.7)
Two to four times a month	456 (21.0)	419 (20.6)	37 (25.9)
Two to three times per week	158 (7.3)	140 (6.9)	18 (12.6)
Four or more times in a week	60 (2.8)	55 (2.7)	5 (3.5)
Not reported	1	1	0
**Tobacco use**			
No	129 (5.9)	117 (5.8)	12 (8.4)
Yes (cigarettes, cigar etc.)	73 (3.4)	65 (3.2)	8 (5.6)
Electronic cigarettes/vaping	52 (2.4)	48 (2.4)	4 (2.8)
Not reported	14 (0.6)	13 (0.6)	1 (0.7)

### Preventive measures used by survey respondents

More than half of the survey respondents (53.1%) used complementary and alternative medicine (CAM) approaches as means of preventive measures (Table 2). Exercises or physical activities (*n* = 396, 50.3 %) were the most commonly used measures, followed by food supplements (*n* = 375, 47.7%) and herbal medicine (*n* = 233, 29.6%). On the other hand, conventional medicine (*n* = 46, 50.6%) was the most commonly used preventive measures among respondents with respiratory symptoms, while those who used CAM approaches mainly used herbal medicines (*n* = 24, 64.9%), followed by home remedies (*n* = 13, 35.1%), and food supplements (*n* = 12, 32.4%). A significant difference was also found between those with respiratory symptoms and those without in the preventive measure used among the CAM approaches. Those with respiratory symptoms preferred using herbal medicine (*p* < 0.001) and home remedies (*p* = 0.001) while those without respiratory symptoms preferred exercising or doing physical activities (*p* = 0.001) as preventive means.

**Table 2:** Preventive approach used by survey respondents.

**Preventive measures**	**Total (*n*, %)**	**Without symptoms (*n*, %)**	**With symptoms (*n*, %)**	***P*-value**
Modern/conventional/chemical medicine	359 (24.2)	313 (22.5)	46 (50.6)	< 0.001*
Other treatments and approaches	787 (53.1)	750 (53.9)	37 (40.7)	0.017*
Anthroposophy	3 (0.4)	3 (0.4)	0 (0)	1.000
Essential oils	20 (2.5)	17 (2.3)	3 (8.1)	0.063
Exercises or activities	396 (50.3)	387 (51.6)	9 (24.3)	0.001*
Food supplements	375 (47.7)	363 (48.4)	12 (32.4)	0.064
Herbal medicine	233 (29.6)	209 (27.9)	24 (64.9)	< 0.001*
Home remedies	114 (14.5)	101 (13.5)	13 (35.1)	0.001*
Homeopathy	2 (0.3)	2 (0.3)	0 (0)	1.000
Special foods and diets	76 (9.7)	72 (9.6)	4 (10.8)	0.774

*p < 0.05, *p*-values were derived from Fisher's exact test.

### Preventive behaviors of survey respondents

Among the respondents' preventive behaviors, the more emphasized behaviors were mask-wearing and hand-washing behavior, where 58.9% of the respondents checked the former and 42.7% checked latter as “Always (or almost).” Those with respiratory symptoms had better preventive behavior in almost all areas (Table 3).

**Table 3:** Preventive behaviors shown by survey respondents.

**Behavior items**	**Category**	**Total (*n*, %)**	**Without symptoms (*n*, %)**	**With symptoms (*n*, %)**
Wash hands with soap or alcohol gel after coming home	Never (or almost)	25 (1.2)	21 (1.0)	4 (2.8)
	Sometimes	204 (9.4)	191 (9.4)	13 (9.1)
	Quite often	535 (24.7)	509 (25.2)	26 (18.2)
	Very often	456 (21.1)	432 (21.4)	24 (16.8)
	Always (or almost)	924 (42.7)	849 (42.0)	75 (52.5)
	Don't know	8 (0.4)	8 (0.4)	0 (0)
	Not applicable	14 (0.7)	13 (0.6)	1 (0.7)
	Missing	11	11	0
Wash hands with soap or gel before eating	Never (or almost)	44 (2.1)	35 (1.8)	9 (6.4)
	Sometimes	349 (16.9)	330 (17.2)	19 (13.5)
	Quite often	499 (24.2)	473 (24.7)	26 (18.4)
	Very often	551 (26.8)	509 (26.5)	42 (29.8)
	Always (or almost)	596 (28.9)	551 (28.7)	0 (0)
	Don't know	13 (0.6)	13 (0.7)	0 (0)
	Not applicable	8 (0.4)	8 (0.4)	0 (0)
	Missing	117	115	2
Maintain social distancing	Never (or almost)	80 (3.8)	75 (3.9)	5 (3.5)
	Sometimes	559 (26.8)	516 (26.5)	43 (30.3)
	Quite often	613 (29.3)	583 (29.9)	30 (21.1)
	Very often	495 (23.7)	461 (23.7)	34 (23.9)
	Always (or almost)	303 (14.5)	274 (14.1)	29 (20.4)
	Don't know	31 (1.5)	30 (1.5)	1 (0.7)
	Not applicable	8 (0.4)	8 (0.4)	0 (0)
	Missing	88	87	1
Consciously avoid touching eyes, mouth or nose	Never (or almost)	110 (5.3)	97 (5.0)	13 (9.2)
	Sometimes	461 (22.3)	426 (22.1)	35 (24.7)
	Quite often	606 (29.3)	566 (29.3)	40 (28.2)
	Very often	493 (23.8)	472 (24.5)	21 (14.8)
	Always (or almost)	355 (17.1)	325 (16.8)	30 (21.1)
	Don't know	37 (1.8)	35 (1.8)	2 (1.4)
	Not applicable	10 (0.5)	9 (0.5)	1 (0.7)
	Missing	105	104	1
Clean things that might have viruses on them	Never (or almost)	332 (15.9)	296 (15.2)	36 (25.5)
	Sometimes	630 (30.2)	585 (30.1)	45 (31.9)
	Quite often	508 (24.4)	479 (24.7)	29 (20.6)
	Very often	374 (18.0)	359 (18.5)	15 (10.6)
	Always (or almost)	203 (9.8)	188 (9.7)	15 (10.6)
	Don't know	24 (1.2)	23 (1.2)	1 (0.7)
	Not applicable	12 (0.6)	12 (0.6)	0 (0)
	Missing	94	92	2
Wear a mask or face covering	Never (or almost)	14 (0.7)	12 (0.6)	2 (1.4)
	Sometimes	124 (6.0)	116 (6.0)	8 (5.6)
	Quite often	282 (13.5)	269 (13.8)	13 (9.2)
	Very often	417 (20.0)	393 (20.2)	24 (16.9)
	Always (or almost)	1,229 (58.9)	1,135 (58.4)	94 (66.2)
	Don't know	12 (0.6)	11 (0.6)	1 (0.7)
	Not applicable	7 (0.3)	7 (0.4)	0 (0)
	Missing	92	91	1
Use any other approaches to try avoiding COVID-19	Never (or almost)	645 (30.5)	599 (30.4)	46 (32.2)
	Sometimes	373 (17.7)	342 (17.4)	31 (21.7)
	Quite often	422 (20.0)	397 (20.2)	25 (17.5)
	Very often	282 (13.4)	267 (13.6)	15 (10.5)
	Always (or almost)	264 (12.5)	244 (12.4)	20 (14.0)
	Don't know	23 (1.1)	22 (1.1)	1 (0.7)
	Not applicable	103 (4.9)	98 (5.0)	5 (3.5)
	Missing	65	65	0
Avoid touching someone else's pets	Never (or almost)	313 (14.9)	301 (15.4)	12 (8.4)
	Sometimes	276 (13.2)	262 (13.4)	14 (9.8)
	Quite often	388 (18.5)	376 (19.3)	12 (8.4)
	Very often	333 (15.9)	318 (16.3)	15 (10.5)
	Always (or almost)	589 (28.1)	519 (26.6)	70 (49.0)
	Don't know	30 (1.4)	28 (1.4)	2 (1.4)
	Not applicable	167 (8.0)	149 (7.6)	18 (12.6)
	Missing	81	81	0
Do a total of 30 min or more of physical activity	0 day	357 (19.5)	306 (18.1)	51 (37.2)
	1 day	288 (15.7)	272 (16.1)	16 (11.7)
	2 days	420 (22.9)	400 (23.6)	20 (14.6)
	3 days	377 (20.6)	352 (20.8)	25 (18.3)
	4 days	126 (6.9)	117 (6.9)	9 (6.6)
	5 days	171 (9.3)	162 (9.6)	9 (6.6)
	6 days	38 (2.1)	37 (2.2)	1 (0.7)
	7 days	54 (3.0)	48 (2.8)	6 (4.4)
	Missing	346	340	6

### Symptoms and treatment measures reported by survey respondents

**Figure 1:**
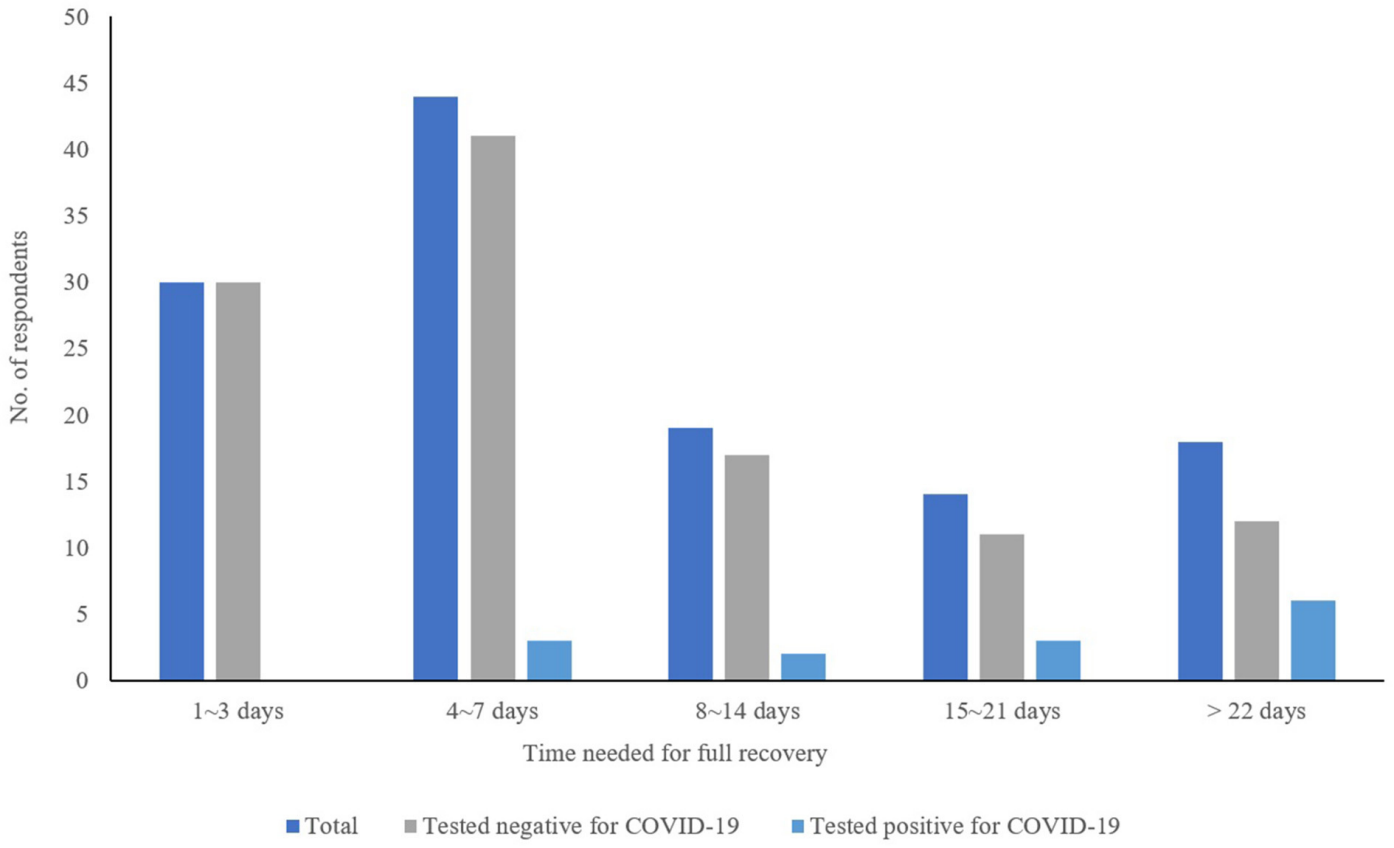
Time needed for full recovery.

The most frequently reported respiratory infections related symptoms were runny or blocked nose (47.6%), cough (45.5%), fever (44.1%), sore throat (42.0%), and fatigue (30.1%) as shown in Table 4. Significant differences were also found between symptoms reported and COVID-19. Those who tested positive for COVID-19 reported higher prevalence of loss of smell or taste (*p* < 0.001) and muscles/joints aches (*p* = 0.016). Most of the respondents improved after 1-week of treatment (*n* = 68, 52.7%) and fully recovered (*n* = 74, 59.2%) within a week (Figure 1).

**Table 4:** Symptoms and treatment measures reported by survey respondents with symptoms, tested COVID-19 positive or negative.

**Category**	**Total (*n*, %)**	**Tested negative for COVID-19 (*n*, %)**	**Tested positive for COVID-19 (*n*, %)**	***P*-value**
**Symptoms reported**				
Headache	46 (32.2)	43 (34.1)	3 (17.7)	0.268
Loss of smell or taste	13 (9.1)	4 (3.2)	9 (52.9)	< 0.001*
New aches and pains in muscles/joints	26 (18.2)	19 (15.1)	7 (41.2)	0.016*
Nausea and/or vomiting	9 (6.3)	7 (5.6)	2 (11.8)	0.290
Pains in your chest	13 (9.1)	10 (7.9)	3 (17.7)	0.187
Runny or blocked nose	68 (47.6)	63 (50.0)	5 (29.4)	0.128
Facial pains, blocked sinus	10 (7.0)	8 (6.4)	2 (11.8)	0.338
Skin rashes	4 (2.8)	3 (2.4)	1 (5.9)	0.401
Sore throat	60 (42.0)	54 (42.9)	6 (35.3)	0.610
Cough	65 (45.5)	55 (43.6)	10 (58.8)	0.302
Coughing up phlegm	41 (28.7)	34 (27.0)	7 (41.2)	0.257
Diarrhea	15 (10.5)	13 (10.3)	2 (11.8)	0.693
Earache	4 (2.8)	4 (3.2)	0 (0)	1.000
Sore eyes	3 (2.1)	3 (2.4)	0 (0)	1.000
Fatigue	43 (30.1)	39 (31.0)	4 (23.5)	0.779
Feeling short of breath	8 (5.6)	6 (4.8)	2 (11.8)	0.243
Fever, high temperature	63 (44.1)	54 (42.9)	9 (52.9)	0.448
**Type of treatment approach**				
Modern/conventional/chemical medicine	81 (62.8)	66 (58.4)	15 (93.8)	0.005*
Other treatments and approaches	30 (23.3)	27 (23.9)	3 (18.8)	0.762
No treatment	34 (26.4)	33 (29.2)	1 (6.3)	0.068
**Outcomes after 1-week treatment**				
Don't know	1 (0.8)	1 (0.9)	0 (0)	0.005*
Getting worse or developed new symptoms	4 (3.1)	2 (1.8)	2 (12.5)	
Improved	68 (52.7)	59 (52.2)	9 (56.3)	
No improvement	13 (10.1)	9 (8.0)	4 (25.0)	
Resolved	43 (33.3)	42 (37.2)	1 (6.3)	
Not reported	14	13	1	
**Duration for full recovery**				
1–3 days	30 (24.0)	30 (27.0)	0 (0)	0.003*
4–7 days	44 (35.2)	41 (36.9)	3 (21.4)	
8–14 days	19 (15.2)	17 (15.3)	2 (14.3)	
15–21 days	14 (11.2)	11 (9.9)	3 (21.4)	
More than 22 days	18 (14.4)	12 (10.8)	6 (42.9)	
Not reported	18	15	3	

*p < 0.05, *p*-values were derived from Fisher's exact test.

### Mental health status of survey respondents

This survey also included questions on mental health conditions which were based on the Patient Health Questionnaire-4 (PHQ-4), a self-report screening scale for anxiety and depression (Table 5). Majority of the respondents (64.9%) did not show signs of mental health issues.

**Table 5:** Mental health conditions of survey respondents based on PHQ-4.

**Category**	**Total (*n*, %)**	**Tested positive for COVID-19 (*n*, %)**	**Tested negative for COVID-19 (*n*, %)**	**Without symptoms (*n*, %)**	**With symptoms (*n*, %)**
**Total PHQ-4**					
Normal	1,412 (64.9)	19 (27.9)	309 (66.0)	1,341 (65.8)	74 (51.7)
Mild	568 (26.1)	43 (63.2)	120 (25.6)	516 (25.3)	53 (37.1)
Moderate	166 (7.6)	2 (2.9)	31 (6.6)	157 (7.7)	9 (6.3)
Severe	31 (1.4)	4 (5.8)	8 (1.7)	24 (1.2)	7 (4.9)
**Anxiety**	223 (10.2)	6 (8.8)	43 (9.2)	202 (9.9)	21 (14.7)
**Depression**	357 (16.4)	8 (11.8)	66 (14.1)	332 (16.3)	26 (18.2)

The total PHQ−4 score complements the subscale scores as an overall measure of symptom burden, functional impairment and disability. Total score is calculated by summing together the scores of each of the 4 items (1. Feeling nervous, anxious or on edge; 2. Not being able to stop or control worrying, 3. Not being able to stop or control worrying, 4. Little interest or pleasure in doing things) in PHQ-4. Scores are rated as normal (0–2), mild (3–5), moderate (6–8), and severe (9–12). Total score ≥3 for first 2 items suggests anxiety. Total score ≥3 for last 2 items suggests depression.

## Discussion

This study demonstrated that the majority of survey respondents had adequate understanding of COVID-19 prevention measures as relevant good practices. We found that individuals with respiratory infection-related symptoms were also more likely to engage in preventive behaviors, such as wearing masks in public places, washing their hands with soap or alcohol gel, and maintaining social distances, indicating health vulnerability affects individual engagement in preventive behaviors.

The majority of respondents in this study had implemented good personal preventive behaviors, where more than 80% of them reported putting on mask when leaving their home. This finding is similar to that of a recent survey in China which reported that 81% of their respondents mentioned wearing a mask in public (15). Another Chinese nationwide study reported that 97.9% of their respondents put on a mask in public and washed their hands more frequently than usual as a preventive measure (13).

However, the mask wearing rate in our finding was higher than other countries. According to a cross-sectional survey, the prevalence of putting on masks was considerably low in Australia (45.5–51.4%) and relatively higher in United Kingdom (70.8%) and United States (75.6–77.4%) (16).

With regard to preventive measures, the more commonly reported measures were exercise or activities, food supplements and herbal medicine. This finding is also similar to a survey study conducted in European countries where more than 50% of the survey respondents from Netherlands and Germany followed a healthy diet or took dietary supplements (14), but in contrast with their findings where only 34.2% practiced regular exercise. Additionally, exercise or physical activities has been found to improve resilience and reduce depressive symptoms (23). Our study adds an additional advantage of exercise or physical activities given its high usage as preventive measures.

Respondents in our survey also rated their mental health as average during the pandemic. Differing from other countries, our respondents did not experience any lockdown measures or extreme restrictions which may somehow reduce the impact of the pandemic on mental health (24). As this survey took place at the early stage of the pandemic, its impact on mental health was relatively small compared to the current status where the pandemic has been ongoing for years. Almost half of the respondents regarded their financial status as concerning during the pandemic. Loneliness and anxiety about money issues had a negative impact on mental health during the pandemic which could have risen due to loss of job, loss of loved ones, quarantine measure, and social distancing (25–27). Therefore, COVID-19 pandemic prevention measures should also include mental health and psychosocial considerations.

The variation in the symptoms reported also indicate that COVID-19 related symptoms often overlap with other respiratory infection related symptoms. Our findings showed that those who tested positive for COVID-19 infection had a significantly higher prevalence of symptoms such as loss of smell or taste, muscle and joint pains which are most representative symptoms of COVID-19 as reported in many previous studies (28–30). Due to the national healthcare policy, conventional medicine was mainly used for the treatment of COVID-19 (31–33). Herbal medicine was also used as treatment and prevention approaches but was not popular (34, 35). Most Korean citizens stored over-the-counter (OTC) conventional medication, such as acetaminophen and ibuprofen, as preventive means that these types of medicines were out of stock from most of the drugstores. Home stocking of OTC medications could also be due to delayed start of the vaccination compared to other countries since Korea started COVID-19 vaccination for the general population around end of August 2021 (36, 37).

However, there are several limitations in this study. The survey was completed purely with the respondents' personal experience and should not be used to infer about the experience or epidemiology of all individuals who had COVID-19 infection. This study was also subjected to selection bias, which may not be entirely representative of the Korean population, as our respondents are mainly in their 30 s and elderly people are under-represented. Besides, this study is based on subjective questioning and the reported symptoms and outcomes were not validated or confirmed.

## Implications

This is the first study to explore the varying physical, psychological symptoms and patient experience during the ongoing COVID-19 pandemic in South Korea. Our findings show that there is a need for differentiated approaches based on individual needs and vulnerability in the engagement in preventive behaviors. We did not investigate on COVID-19 vaccination since no vaccine was available at the time of data collection; therefore, future studies could also explore the association between COVID-19 vaccination and different preventive health behaviors. Future research may also take pandemic-related factor such as vaccination doses or long COVID into account as they could refine our understanding on the impact of pandemic on general population. Future longitudinal studies and interventions would be needed to provide more insights to improve the management of pandemic in the long run.

## Conclusion

In South Korea, conventional medicine was mainly used for COVID-19 treatment whereas CAM was commonly used as preventive measures. COVID-19 was also found to have less impact on the general population's mental health. The findings of this study may shed light on how the pandemic impacted the general population. Future COVID-19 and pandemic planning should take individual's experiences into consideration.

## Data availability statement

The raw data supporting the conclusions of this article will be made available by the authors, on reasonable request.

## Ethics statement

The studies involving human participants were reviewed and approved by Kyunghee University [KHSIRB-20-574 (EA), November 2020] and University of Southampton (ERGO 56975, May 2020). The patients/participants provided their written informed consent to participate in this study.

## Author contributions

This work is conceptualized by MW, BG, JH, X-YH, and ML. The study methodology was designed by MW, BG, JH, and X-YH validated by HL, T-HK, HWL, and ML. Formal analysis and data curation were performed by MY and LA. The original draft was written by LA, ES, and MY and reviewed by J-WL, YJ, JK, EK, YP, HWL, and ML. This project was supervised and administered by ML. All authors contributed to the article and approved the submitted version.

## Funding

This work was supported by Korea Institute of Oriental Medicine (KSN20224112). This funding source did not participate in the design of this study or play any role during its execution, analyses, interpretation of the data, manuscript drafting, or decision to submit results.

## Conflict of interest

The authors declare that the research was conducted in the absence of any commercial or financial relationships that could be construed as a potential conflict of interest.

## Publisher's note

All claims expressed in this article are solely those of the authors and do not necessarily represent those of their affiliated organizations, or those of the publisher, the editors and the reviewers. Any product that may be evaluated in this article, or claim that may be made by its manufacturer, is not guaranteed or endorsed by the publisher.
